# Pulsed Ultrasound-Assisted Extraction as an Alternative Method to Conventional Maceration for the Extraction of the Polyphenolic Fraction of *Ribes nigrum* Buds: A New Category of Food Supplements Proposed by The FINNOVER Project

**DOI:** 10.3390/foods8100466

**Published:** 2019-10-10

**Authors:** Federica Turrini, Dario Donno, Gabriele Loris Beccaro, Paola Zunin, Anna Pittaluga, Raffaella Boggia

**Affiliations:** 1 Department of Pharmacy, University of Genoa, Viale Cembrano 4, 16148 Genoa, Italy; turrini@difar.unige.it (F.T.); zunin@difar.unige.it (P.Z.); pittalug@difar.unige.it (A.P.); boggia@difar.unige.it (R.B.); 2 Department of Agriculture, Forestry and Food Science, University of Torino, Largo Braccini 2, 10095 Grugliasco (TO), Italy

**Keywords:** phenolic compounds, bud derivatives, *Ribes nigrum* glyceric macerate, green chemistry, pulsed ultrasound-assisted extraction, untargeted spectroscopic fingerprint, targeted chromatographic fingerprint

## Abstract

In this research, for the first time, an alternative method to produce *Ribes nigrum* bud derivatives is presented. Pulsed ultrasound-assisted extraction (PUAE), using a food-grade solvent according to green chemistry principles, has been employed and compared to the conventional extraction method. Traditionally, bud derivatives, a category of botanicals marketed as plant food supplements in the European Community, are produced by macerating meristematic tissues of trees and plants mainly spontaneously collected. Buds are a challenging raw material for the UAE, since meristematic tissues are much softer and fragile than their corresponding adult phenological stage. It is therefore important to assess whether the polyphenolic fraction, very susceptible to degradation, is conserved after UAE. Untargeted polyphenolic fingerprints (UV-Visible and fluorescence) coupled with chemometrics are employed to quickly screen the best extraction conditions, evaluated by the design of experiment (DoE) method. The polyphenolic fraction of the optimized PUAE extract was quantified by targeted HPLC fingerprint and its antiradical activity was determined. PUAE on a lab pilot reactor was proven to be the most practical approach for a rapid (20 min vs. 21 days maceration) and efficient extraction of bioactive polyphenolics from *Ribes nigrum* buds, encouraging the scale up to an industrial plan.

## 1. Introduction

FINNOVER “Innovative strategies for the development of crossborder green supply chains” is an Interreg ALCOTRA Italy/France transfrontier cooperation project (2017–2020) whose main target is the “green” implementation of some agro-industrial chains [1]. Particularly, the project supports the creation of both innovative and eco-sustainable production chains of botanicals in order to valorize the biodiversity of the ALCOTRA territory. One of the main natural products studied in FINNOVER are bud derivatives, which represent a relatively new category of botanicals marketed, in the European Community, as plant food supplements according to the Directive 2002/46/EC of the European Parliament [2,3]. Bud derivatives are obtained by cold maceration in solvents (i.e., ethanol and glycerol) of fresh meristematic tissues of trees and plants (i.e., buds and young sprouts) as reported in the European Pharmacopoeia VIII edition (2014) [2,4,5]. These botanicals are still poorly studied, although they are widely used in gemmotherapy, a branch of phytotherapy that exploits the properties of these plant extracts for medicinal purposes [6]. The peculiarity of meristematic tissues in this particular phenological stage concerns their fragile texture and the high content of compounds which constitute the bud phytocomplex. In fact, these substances, including mainly flavonoids, enzymes, vitamins, aminoacids, nucleic acids, and plant hormones, are often present only in trace in the corresponding adult tissues [5].

In this research, a sonochemical application with a green chemistry approach was presented. Particularly, pulsed ultrasound-assisted extraction (PUAE) was employed as alternative method to quickly produce new bud derivatives in comparison to the long traditional maceration in solvent (21 days) taking under control their total phenolic fraction and antiradical activity to monitor possible PUAE-induced degradations. *Ribes nigrum* buds (RNB) were used as case study due to their common use in herbal medicine for their potential health properties. The most important industrial products of *R. nigrum* are its berries, which contain very high amounts of bioactive compounds, particularly flavonoids, phenolic compounds, and anthocyanins [7,8,9]. However, *R. nigrum* bud derivatives also contain high amounts of polyphenols, representing more than 60% of the bud phytocomplex [7], and they are widely used for inflammatory, circulatory, respiratory, and cutaneous disorders [10].

Ultrasounds (UAE—ultrasound-assisted extraction) together with microwaves (MAE—microwave-assisted extraction), supercritical fluids (SCF), and pulsed electric fields (PEF), are emerging “green” extraction technologies [11]. According to the six principles of the green extraction introduced by Chemat and colleagues [12] and to the twelve principles of green chemistry set by the Environmental Protection Agency of USA [13], these eco-compatible extraction techniques, with respect to conventional methods, aim to reduce the environmental impact in terms of time and energy. Moreover, they reduce both the quantities of solvents employed, preferring alternative solvents (water or food-grade solvents), and the generation of waste, hazardous substances, and consequently pollution [11,14]. In particular, UAE is a relatively simple, cheap, and efficient alternative to conventional extraction techniques whose main benefits are faster kinetics and increased extraction efficiency [15,16]. In fact, UAE allows one to quickly extract, with high reproducibility both on small and large scale, a wide variety of bioactive compounds (i.e., aromas, pigments, antioxidants, and organic and mineral compounds) from several animal tissues, plants, or food matrices [15,17]. However, the effects of ultrasound on the extraction yield may be linked to the nature of the matrix. Therefore, the experimental conditions of UAE must be optimized for each matrix [18]. In a solid/liquid media, the ultrasound waves originate the cavitation phenomena, a succession of different phases of compression and rarefaction which generates cavitation bubbles in the liquid. The implosion of the cavitation bubbles on the surface of the solid material generate microjets, at very high temperature and pressure, which destroy the wall cells of the matrix, with the consequent recovery of the intracellular content in the extraction solvent. There are several mechanisms involved (i.e., fragmentation, erosion, sonoporation, detexturation, capillarity) which independently or in combination influence the final ultrasound extraction yield [17]. When UAE is used in a pulsed mode (PUAE), the ultrasound processor works intermittently during the entire extraction process (active time vs. inactive time). This extraction mode reduces the operating temperature, allowing the extraction of thermolabile compounds and decreasing the possibility to produce alterations (i.e., oxidation products) in the final extract [19].

Untargeted phytochemical fingerprints coupled with chemometrics [2,20], particularly the UV-Visible and fluorescence spectra of each extract as multivariate response variables, have been employed to quickly screen the best experimental conditions of PUAE investigated by the design of experiment (DoE) method [21]. Finally, the extract obtained by PUAE in the optimized conditions has been characterized using targeted phytochemical fingerprinting by HPLC [2,22,23] in order to identify and quantify the main polyphenolic compounds. The polyphenolic fraction has been selected as the marker of activity and degradation susceptibility in order to make a comparison with the corresponding *R. nigrum* glyceric macerate (RNGM), representing the commercial product.

## 2. Materials and Methods

### 2.1. Bud Collection

RNB were collected from plants cultivated in the Bronda valley (Cuneo, Italy), particularly in the municipality of Pagno (44.597,7.424–44.598,7.424) and Brondello (44.603,7.422–44.603,7.418) in March 2018. Buds, after being certified by a botanical expert, were employed by an Italian Company of food supplements (Geal Pharma, Bricherasio, Turin, Italy) for the production of the corresponding RNGM. Particularly, the fresh merystematic tissues were immediately used after their collection in order to preserve their bioactive compounds. Both the traditional procedure for preparing glyceric macerates, according to the European Pharmacopoeia VIII edition, and an alternative method that exploits the action of ultrasounds have been used and compared for the production of the corresponding extracts (RNGM and RN8).

### 2.2. Chemicals

Ethanol and glycerol were supplied by VWR International S.r.l (Milan, Italy) and GealPharma (Bricherasio, Turin, Italy), respectively. All the standards employed for the HPLC analysis were purchased by Sigma-Aldrich (St. Louis, MO, USA). The purity of all the standards employed was ≥95%. Ultrapure water (18 MΩ) was produced by a Millipore Milli-Q system (Bedford, MA, USA) and used throughout.

### 2.3. Traditional Preparation of R. nigrum Glyceric Macerates

RNGM was produced according to the indications of the European Pharmacopoeia VIII edition (2014), referring to the procedure reported in the French Pharmacopoeia [4]. Particularly, a mixture of glycerol/ethanol 96% (1:1 *w*/*w*) as extraction solvent and a solid–solvent ratio 1:20 between buds and solvent (considering the dry weight) were employed. The phytocomplex extraction from RNB involved several steps: a cold maceration for 21 days, followed by a preliminary filtration, a manual pressing, and, at the end, a second filtration with filter paper (Whatman n. 1) after 2 days of decanting [2,5]. The obtained extracts, which represent the commercial product marketed by GealPharma, were stored at 4 °C in the dark until further analysis.

### 2.4. Alternative Method to Produce R. nigrum Bud Derivatives: Pulsed Ultrasound-Assisted Extraction (PUAE)

PUAE was carried out directly by an Hielscher UP200St (Teltow, Germany) in pulsed mode, with an ultrasonic titanium probe (7 mm diameter) able to transfer, with high efficiency, the acoustic energy into the treated media [2,24,25]. Fresh RNB were finely ground by a Grindomix 200 M (Retsch, Haan, Germany), for 20 s at 5000 rpm, and then sieved by a 150 µm sieve. Twenty grams of a glycerol/ethanol 96% mixture 1:1 *w*/*w* were added to 1 g (dry weight) of ground RNB in a polypropylene 50 mL centrifuge tube. The samples were processed in a 200 W ultrasonic processor at a constant frequency of 26 kHz, with an amplitude level of 30%, optimized in a previous paper from the authors [26], keeping temperature under control always below 70 °C. The pulse duration and pulse interval refer to ‘‘on’’ and ‘‘off’’ times of the sonicator. The same mixture of glycerol/ethanol 96% (1:1 *w*/*w*) and the same solid–solvent ratio 1:20 between buds and solvent (considering the dry weight), as described in the Section 2.3, were employed. The duty cycle (pulse) and the extraction time (65% and 20 min, respectively) were optimized by applying DoE (Table 1). The obtained suspension was then filtered by Buchner (Whatman n. 44 paper) and the filtrate was centrifuged at 3000 rpm for 10 min. The obtained solutions were stored at 4 °C until the analysis time.

### 2.5. Untargeted Fingerprints of The R. nigrum Phytocomplex

#### 2.5.1. UV-Visible Spectroscopy

An Agilent UV-Vis spectrophotometer Cary 100 (Varian Co., Santa Clara, CA, USA) with 0.5 nm resolution, was employed to record all the UV-Vis spectra of the extracts and the corresponding glyceric macerates of *R. nigrum*. Before being analyzed, all the samples were properly diluted 1:20 in the same extraction mixture (glycerol/ethanol 96% 1:1 *w*/*w*). The total spectrum of each analyzed sample was collected in duplicate at room temperature (25 ± 1 °C), against a blank solution (i.e., the extraction mixture), using rectangular quartz cuvettes with 1 cm path length. For each sample, the resulting spectra were averaged and used as vector of variables to build the data matrix.

#### 2.5.2. Fluorescence Spectroscopy

The excitation–emission fluorescence spectra were recorded in duplicate at room temperature (25 ± 1 °C) by a Perkin-Elmer LS55B luminescence spectrometer (Waltham, MA, USA) using the traditional right angle fluorescence spectroscopic technique [27]. A standard cell holder and a 10 mm quartz SUPRASIL^®^ cell with volume of 3.5 mL by PerkinElmer were used. The emission spectra were recorded in the range of 450–800 nm, exciting samples at a fixed wavelength (λ ex = 430 nm) [20]. Both the excitation and the emission monochromator slits were set to 10 nm, with high gain and 600 nm/min of speed. The same dilution of all the samples, at the ratio of 1:20 with the solvent, was evaluated. For each sample, the resulting emission spectra were averaged and used as vector of variables to build the data matrix combing them together with the previous described UV-Vis spectra.

### 2.6. Experimental Design and Multivariate Data Analysis

DoE was used to optimize the experimental conditions of PUAE from RNB. A Faced Central Composite Design (2k + 2^k^ + 1) was applied with the aim to estimate the constant, the linear terms, the interactions between variables, and the quadratic terms, according to the following model [21]:

Y = b_0_ + b_1_X_1_ + b_2_X_2_ + b_12_X_1_X_2_ + b_11_X_1_^2^ + b_22_X_2_^2^

The experimental plan, illustrated in Table 1, summarizes the conditions of the nine experiments performed (namely from RN1 to RN9). The minimum, intermediate, and maximum value of each variables are labeled as −1, 0, and +1, respectively. A data matrix M_9,1402_ consisting of nine rows (the nine samples/extracts obtained by DoE) and 1402 columns (the vector of 701 absorbance values at different wavelengths in the range of 230–500 nm of the UV-Vis spectra plus 701 fluorescence emissions in the range of 450–800 nm of the fluorescence spectra), was prepared and further analyzed by the PCA, a multivariate statistical technique of unsupervised pattern recognition. The scores on PC1 have been used as a response variable of the experimental design (Y). In detail, the standard normal variate (SNV) transform, or row autoscaling, was previously performed on the spectral data in order to revise both the baseline shifts and the global intensity variations [28]. Subsequently, PCA was performed using the nonlinear iterative partial least squares (NIPALS) algorithm on the column-centered data [29]. After the PCA, the scores on PC1, explaining the 90.3% of the total variance, were extracted and used as response to elaborate DoE. DoE and multivariate data analysis were performed by CAT (Chemometric Agile Tool) a chemometric software based on R, developed by the Chemistry Group of the Italian Chemical Society [30]. The data matrix and the detailed PCA analysis is available as Supplementary Materials.

### 2.7. Analytical Determinations

The most promising extract obtained by PUAE and the corresponding glyceric macerate (RNGM), in order to make a comparison, were characterized to evaluate their total phenolic contents (TPC) and their radical scavenging activity (RSA). All the measurements were performed in duplicate and the results are expressed as mean ± standard deviation (SD). Statistical analysis was performed by the Excel Data Analysis Tool (Microsoft Corporation, Seattle, WA, USA).

#### 2.7.1. Determination of The Total Phenolic Compounds (TPC)

The Folin-Ciocalteu spectrophotometric method was applied to estimate the TPC of the *R. nigrum* bud preparations [31]. 0.2 mL of sample appropriately diluted, 1 mL of Folin-Ciocalteu reagent (diluted 1:10 with deionized water), 0.8 mL of aqueous sodium carbonate 7.5% *w*/*v* solution were added in a test tube and vortexed. After an incubation period of 30 min at room temperature in the dark, the absorbance was recorded at 760 nm by an Agilent 8453 UV-Vis spectrophotometer with 1 nm resolution. A calibration curve, using gallic acid as a standard, has been used to evaluate the polyphenolic concentration. The TPC was expressed as milligrams of gallic acid equivalent (GAE) pulled-out from 100 mL of bud extract (mg GAE/100 mL).

#### 2.7.2. Determination of Radical Scavenging Activity (RSA)

The DPPH• assay was applied to evaluate the RSA of the *R. nigrum* bud preparations [32]. Determinations were performed as described in a previous paper [24]. The absorbance at 515 nm was recorded by an Agilent 8453 UV-Vis spectrophotometer with 1 nm resolution. A multilevel calibration with ascorbic acid as standard was used to evaluate the RSA and to express the results as milligrams of ascorbic acid equivalent (AAE) in 100 mL of bud extract (mg AAE/100 mL).

### 2.8. HPLC Analysis

HPLC methods were used for phytochemical analysis both on *R. nigrum* bud preparations and PUAE extracts. Analysis were focused on flavonols, phenolic acids (benzoic and cinnamic acids), and catechins, as polyphenolic markers with a demonstrated health-promoting activity [33]. Bioactive compounds were identified and quantified by comparison and combination of their retention times and UV spectra with those of authentic standards. The calibration parameters for all the employed analytical standards were previously reported by the authors [22,34]. The total bioactive compound content (TBCC) was determined as sum of the selected and identified markers with health-promoting activities and positive antioxidant effects on human health-status according to “multimarker approach” [35]: phytochemicals were grouped into different bioactive classes in order to evaluate each class contribution to phytocomplex composition. All analyses were triplicated and the results expressed as mg/100 g of fresh weight (FW).

Samples were filtered with circular pre-injection filters (0.45 µm, polytetrafluoroethylene membrane) prior to HPLC-DAD analysis. Chromatographic analysis was carried out using an Agilent 1200 High-Performance Liquid Chromatograph coupled to an Agilent UV-Vis diode array detector (Agilent Technologies, Santa Clara, CA, USA), based on HPLC methods previously validated for fresh fruits, herbal medicines, and other food products [2,22,23]. Chromatographic conditions were set in order to obtain a phytochemical information with a good resolution and a reasonable analysis time.

Bioactive molecule separation was achieved on a Kinetex C18 column (4.6 mm × 150 mm, 5 μm, Phenomenex, Torrance, CA, USA). Different mobile phases were used for bioactive compound characterization and several linear gradients in different slopes were optimized because some compounds were similar in structure with each other in the same chemical class: (1) a solution of 10 mM KH_2_PO_4_/H_3_PO_4_ and acetonitrile with a flow rate of 1.5 mL·min^−1^ (method A—analysis of cinnamic acids and flavonols); (2) a solution of methanol/water/formic acid (5:95:0.1 *v*/*v*/*v*) and a mix of methanol/formic acid (100:0.1 *v*/*v*) with a flow rate of 0.6 mL·min^−1^ (method B—analysis of benzoic acids and catechins). Selected wavelengths were suitable to achieve more specific peaks as well as a smooth baseline after a full scan on the chromatogram from 190 to 400 nm; in particular, UV spectra were recorded at 330 nm (A) and 280 nm (B). Information on used chromatographic methods and selected markers are reported in the Supplementary material.

## 3. Results and Discussion

### 3.1. Optimization of The PUAE Experimental Conditions by DoE Using Untargeted Phytochemical Fingerprint

The PUAE conditions have been optimized by a Faced Central Composite Design (CCD), whose results are shown in Table 1. The DoE response variable to be optimized was obtained by an untargeted spectroscopic method combined with chemometrics previously described by the authors [2,20]. Briefly each of the nine extracts, obtained according the experimental plan and spectroscopic analyzed, was described by a vector of 701 UV-Vis absorbances plus 701 fluorescence emissions, as a holistic nontargeted fingerprint 1402-dimensional of the corresponding extract. Since these multivariate vectors of UV-Vis absorptions and fluorescence emissions (701 + 701 variables) of each extract have been proven to be strictly correlated to the whole polyphenolic fraction of the extracts they were combined in a multivariate data matrix: The DoE response matrix. This matrix, composed of nine rows and 1402 columns (M_9,1402_: nine objects corresponding to the nine experiments and 1402 variables which represent the spectral absorptions/emissions), has been elaborated by principal component analysis (PCA), an unsupervised patter recognition technique, in order to extract the useful analytical information and to reduce its dimensionality.

**Figure 1:**
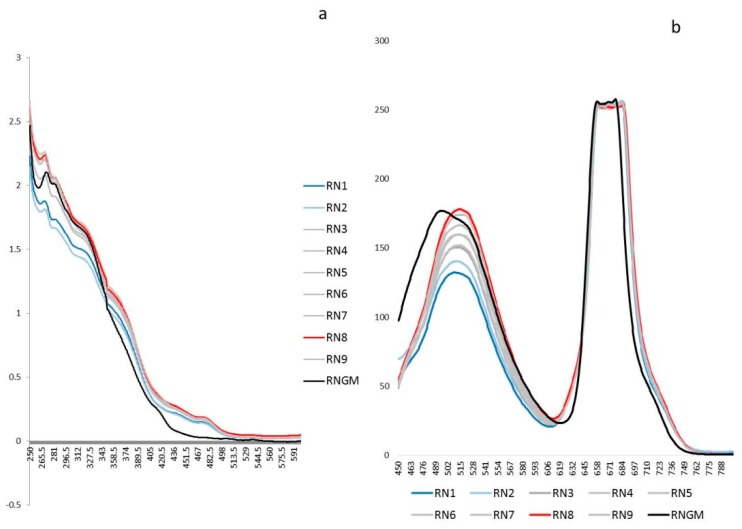
Untargeted spectroscopic fingerprints of the *R. nigrum* bud (RNB) phytocomplex: (**a**) The UV-Vis averaged spectra (250–600 nm) of the nine experiments selected by the DoE and the corresponding *R. nigrum* glyceric macerate (RNGM) tested at the same dilution (1:20 in the extraction solvent); (**b**) The 2D Fluorescence averaged spectral emissions (450–800 nm) of the nine experiments selected by the DoE and the corresponding RNGM tested at the same dilution (1:20 in the extraction solvent).

The untargeted polyphenolic phytochemical fingerprints (UV-Vis absorptions and Fluorescence emissions) of each extract obtained by DoE were reported in Figure 1 and compared with the corresponding commercial product RNGM.

**Figure 2:**
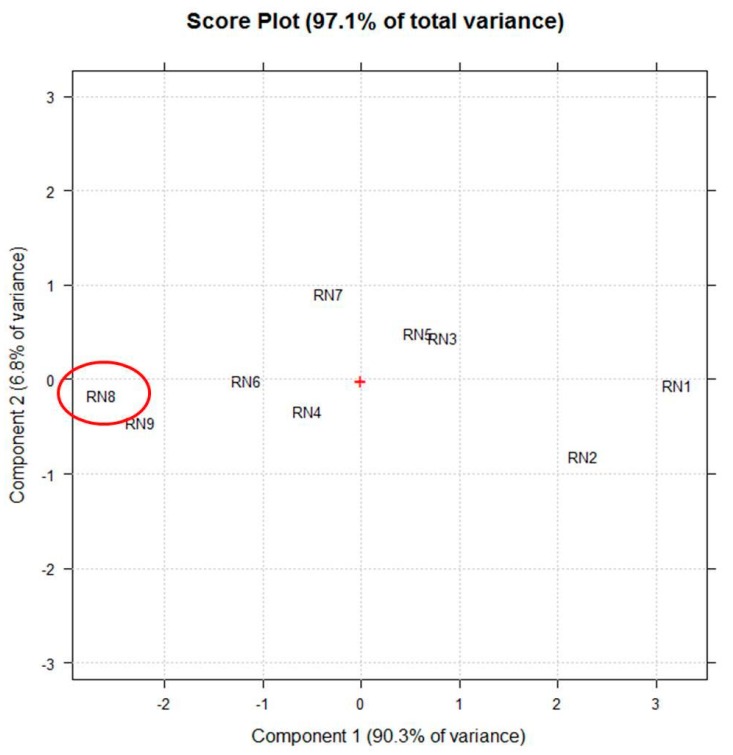
The score plot on the first two principle components (PCs) selected by principle component analysis (PCA) using the vector of UV-Vis (250–600 nm) spectra coupled to fluorescence (450–800 nm) absorptions of each extract (RN1–RN9) as a multivariate untargeted signal. The red + represents the central point of the plot and the red circle highlights the extract obtained in the best experimental conditions.

Figure 2 shows the score-plot on the first two principal components (PCs), whose explained variance are 90.3% and 6.8%, respectively.

The first PC (PC1) retains all the useful information of the 1402 original variables and thus the corresponding scores were used as the response variable of each experiment in the experimental matrix. The other PCA details are reported in the Supplementary Materials (i.e., score matrix, loading matrix, eigenvalues, explained variance plot). The following model of the CCD has been obtained by applying multiple linear regression to the experimental matrix:

Y = −2.6708 − 0.6814X_1_ * − 1.2487X_2_ ** − 0.1010X_1_X_2_ + 2.6000X_1_^2^ ** 1.4061X_2_^2^ *

* indicates the significance of the coefficients: * = *p* < 0.05, ** = *p* < 0.01.

**Figure 3:**
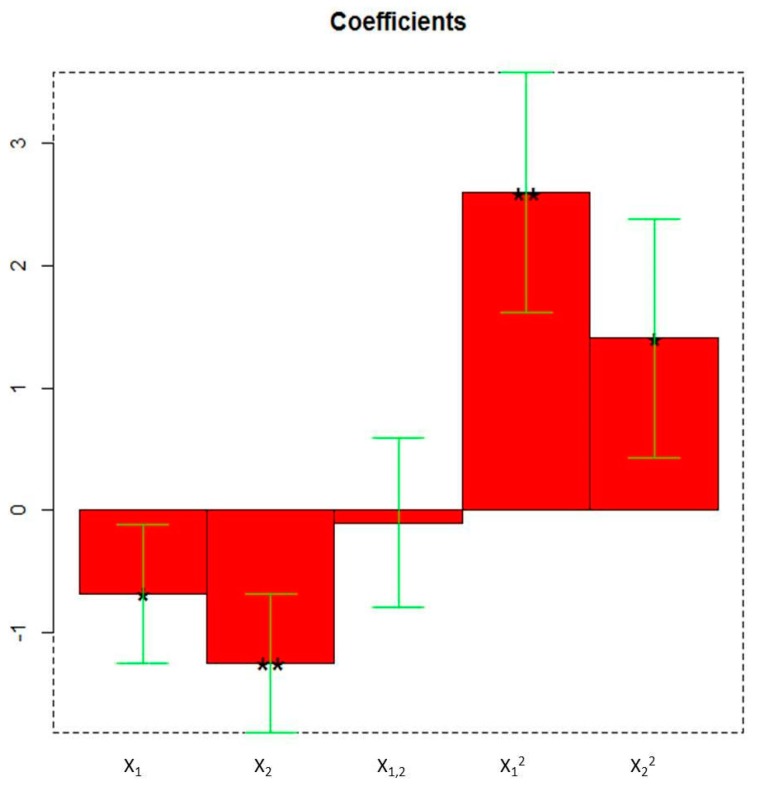
The coefficients of the model of Y (PC1 scores) obtained by the Faced Central Composite Design (X_1_: duty cycle; X_2_: extraction time). * = *p* < 0.05, ** = *p* < 0.01.

All the linear and quadratic terms are significant as highlighted in the plot of the coefficients (Figure 3).

Particularly, the linear term X_2_ (** = *p* < 0.01) corresponding to the extraction time, the quadratic term X_1_^2^ (** = *p* < 0.01), the linear term X_1_ (* = *p* < 0.05) corresponding to the duty cycle, and the quadratic term X_2_^2^ (* = *p* < 0.05) are the statistically significant coefficients. They should be increased or, on the contrary, decreased to improve or to minimize the Y response variable respectively. Experiments whose scores on PC1 are negative (Figure 2) correspond to the highest absorptions/emissions of the phytocomplex as shown in Figure 1, thus they must be decreased. RN8 represents the best experimental conditions and this extract has been analytically characterized and compared with RNGM.

### 3.2. Analytical Characterization of The Most Promising PUAE Extract (RN8) and the Corresponding RNGM

#### 3.2.1. Determination of the Total Phenolic Compounds (TPC) and the Radical Scavenging Activity (RSA)

RN8, representing the most promising *R. nigrum* extract obtained by PUAE, and the corresponding RNGM, were analytically characterized to evaluate their total phenolic contents (TPC) and their radical scavenging activity (RSA). As reported in Table 2, both the bud preparations presented quantitatively similar RSA values: 1158.58 ± 73.24 mg/100 mL of bud extract for RN8 and 1137.04 ± 38.49 mg/100 mL of bud extract for RNGM, respectively. Regarding TPC, RN8 showed a higher value with respect to RNGM (415.56 ± 5.52 mg/100 mL vs. 276.44 ± 3.85 mg/100 mL). However, the Folin-Ciocalteu assay is a nonspecific method to quantify phenols and polyphenols. In fact, this reagent does not measure only phenols, but can react with some reducing substances (i.e., ascorbic acid) [36]. For this reason, the phenol content could be overestimated and further investigations on the phytocomplex composition should be carried out. Nevertheless, the higher value of RN8 is promising and indicative of almost no oxidative alterations potentially induced by ultrasounds with respect to maceration.

#### 3.2.2. Targeted Phytochemical Fingerprint

Antioxidant compounds (in particular, polyphenols) may play a critical health-promoting role in humans for disease prevention due to their synergistic or additive biological effects (phytocomplex) that influence human health better than a single molecule or a group of few compounds [37]. In this study, 13 biologically active compounds (grouped into four polyphenolic classes) were selected as markers for fingerprint analysis because they have been described as important health-effective substances in humans [38]. The phytochemical fingerprints of RNGM and RN8 are reported in Table 3.

Among the analyzed compounds, chlorogenic acid, ferulic acid, hyperoside, and isoquercitrin were not detected. Bioactive compounds were separated and identified via HPLC-DAD. Adding other markers with demonstrated biological activity may be a necessary step for a better identification of the chromatographic pattern in further fingerprint studies together with a mass spectrometry detection of unknown peaks.

**Figure 4:**
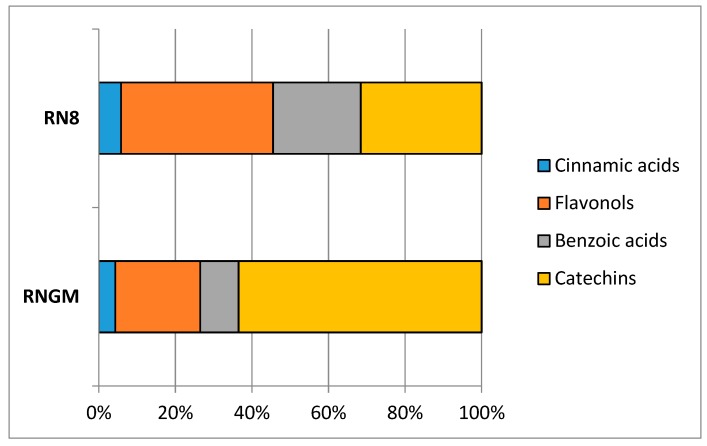
Contribution of each polyphenolic class to the total phytocomplex in RN8 and RNGM analyzed samples.

In RNGM samples, catechins were the most important bioactive class (44.36%), with flavonols as the second most abundant (27.82%), followed by benzoic acids and cinnamic acids (19.93% and 7.89%, respectively), while in PUAE extracts RN8, the quantitative relationships between catechins and flavonols were reversed: 29.81% for catechins and 42.39% for flavonols, while cinnamic acids (6.20%) and benzoic acids (21.60%) showed percentages similar to the bud macerates (Figure 4).

**Figure 5:**
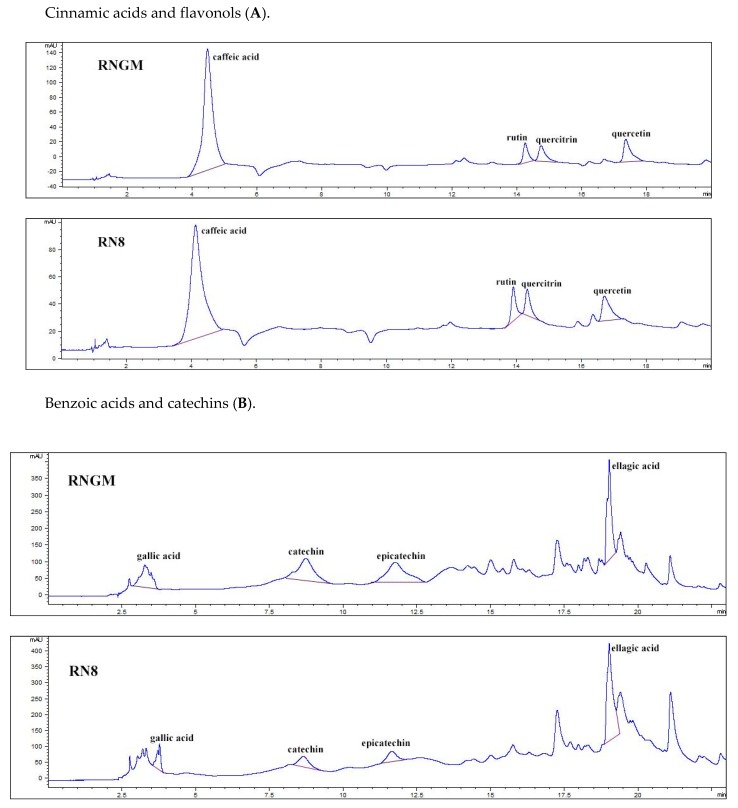
Chromatographic pattern of polyphenolic compounds identified in RN8 and RNGM samples: (**A**) Cinnamic acids and flavonols; (**B**) Benzoic acids and catechins. In the chromatograms, the x-axis represents the signal current intensity (mAU), while the y-axis represents time (min).

Figure 5 reports the polyphenolic chromatographic profile of analyzed samples: RNGM and RN8 presented qualitatively and quantitatively similar phenolic patterns. The most important differences were only detected in four compounds: (i) quercetin (48.53 ± 0.49 mg/100 g of bud fresh weight, FW, for RNGM and 80.14 ± 1.08 mg/100 g FW for RN8); (ii) quercitrin (30.86 ± 0.85 mg/100 g FW for RNGM and 48.18 ± 0.94 mg/100 g FW for RN8); (iii) catechin (95.88 ± 0.26 mg/100 g FW for RNGM and 55.85 ± 2.78 mg/100 g FW for RN8); (iv) epicatechin (59.83 ± 0.37 mg/100 g FW for RNGM and 49.08 ± 0.48 mg/100 g FW for RN8). Caffeic acid showed levels slightly higher in RNGM, while ellagic acid presented levels slightly higher in RN8.

Our results highlight that the traditional glyceric macerate and the alternative PUAE extract show similar total polyphenolic levels (and a qualitatively similar chromatographic pattern), but some differences in specific bioactive compounds (in particular, flavonols and catechins) were also detected, due to the different extraction method [39]. For this reason, PUAE yielded an extract rich in biological active molecules with potentially high health-promoting activity, but maybe with a practical use which could be different from traditional bud preparations. In any case, this research is only a preliminary study and further phytochemical, clinical, toxicological, and pharmaceutical in vitro and in vivo tests should be carried out to confirm this preliminary hypothesis.

## 4. Conclusions

In this work, PUAE, as an alternative time-saving method to produce *R. nigrum* bud derivatives, was presented according to the green chemistry principles. The unconventional extraction conditions were optimized by DoE at the lab scale using untargeted fingerprints coupled to chemometrics, but this same quick strategy could be analogously applied to transfer this method to an industrial scale. The impact of the UAE with respect to traditional maceration was evaluated in terms of recovering of the total phenolic content, the antiradical scavenging activity and the profiles of the most important bioactive compounds. In particular, PUAE provided the extract named RN8 in a few minutes, compared to the 21 day-long maceration, whose total polyphenolic levels and antiradical scavenging are similar or even slightly increased with respect to RNGM. Furthermore, RN8 presents a qualitatively similar chromatographic pattern, even if some differences in flavonols and catechins were detected. Due to these differences, an LC-MS study of RN8 is mandatory in the near future. Nevertheless, this is a promising preliminary result to provide alternative uses of *Ribes nigrum* bud derivatives using this unconventional time-saving extraction method.

## Figures and Tables

**Table 1:** Experimental matrix of the Faced Central Composite Design, the experimental plan (in brackets), and the obtained response variable (Y).

Experiment	Experimental Conditions		Response Variable
	X_1_ Duty Cycle (%)	X_2_ Extraction Time (min)	Y PC_1_ Scores
RN1	−1 (50)	−1 (10)	3.222515159
RN2	+1 (80)	−1 (10)	2.256792019
RN3	−1 (50)	+1 (20)	0.834250201
RN4	+1 (80)	+1 (20)	−0.535350397
RN5	−1 (50)	0 (15)	0.587361402
RN6	+1 (80)	0 (15)	−1.165491045
RN7	0 (65)	−1 (10)	−0.327200585
RN8	0 (65)	+1 (20)	−2.638815847
RN9	0 (65)	0 (15)	−2.234060907

**Table 2:** Total phenolic compounds (TPC) and radical scavenging activity (RSA) of the most promising *R. nigrum* extract obtained by pulsed ultrasound-assisted extraction (PUAE) (RN8) compared to the corresponding commercial product (RNGM).

Determination		RNGM		RN8	
		Mean Value	SD	Mean Value	SD
TPC	*mg GAE*/*100 mL bud extract*	276.44	3.85	415.56	5.52
*RSA*	*mg AAE*/*100 mL bud extract*	1137.04	38.49	1158.58	73.24

Results are reported as mg/100 mL of bud extract and expressed as mean value ± standard deviation (SD) (*n* = 2). GAE: gallic acid equivalent; AAE: ascorbic acid equivalent.

**Table 3:** Targeted phytochemical fingerprint by HPLC-DAD of the polyphenolic compounds in the most promising *R. nigrum* extract (RN8) obtained by PUAE compared to the corresponding glyceric macerate (RNGM).

Bioactive Class	Compound	RNGM		RN8	
		Mean Value	SD	Mean Value	SD
		(mg/100 g FW)		(mg/100 g FW)	
Cinnamic acids	*caffeic acid*	22.48	0.04	20.76	0.48
	*chlorogenic acid*	n.d.	/	n.d.	/
	*coumaric acid*	5.21	0.15	1.05	0.25
	*ferulic acid*	n.d.	/	n.d.	/
Flavonols	*hyperoside*	n.d.	/	n.d.	/
	*isoquercitrin*	n.d.	/	n.d.	/
	*quercetin*	49.53	0.49	80.14	1.08
	*quercitrin*	30.86	0.85	48.18	0.94
	*rutin*	17.25	0.22	20.88	0.48
Benzoic acids	*ellagic acid*	69.66	0.08	75.37	0.30
	*gallic acid*	0.31	0.09	0.64	0.05
Catechins	*catechin*	95.88	0.26	55.85	2.78
	*epicatechin*	59.83	0.37	49.08	0.48

Results are reported as mg/100 g of bud fresh weight (FW) and expressed as mean value ± standard deviation (SD) (*n* = 3). * n.d. = not detectable.

## Supplementary Materials

Supplementary materials are available online at https://www.mdpi.com/2304-8158/8/10/466/s1. Table S1: score matrix; Table S2: loading matrix; Table S3: eigenvalue matrix; Table S4: chromatographic conditions. Figure S1: percentage of explained variance plot.
