# Supplementary figures and images for: Pulsed Ultrasound-Assisted Extraction as an Alternative Method to Conventional Maceration for the Extraction of the Polyphenolic Fraction of Ribes nigrum Buds: A New Category of Food Supplements Proposed by The FINNOVER Project
Source: Foods. 2019 Oct 10;8(10):466. doi: 10.3390/foods8100466 (PMC6835381; doi:10.3390/foods8100466)

**Figure S1.**


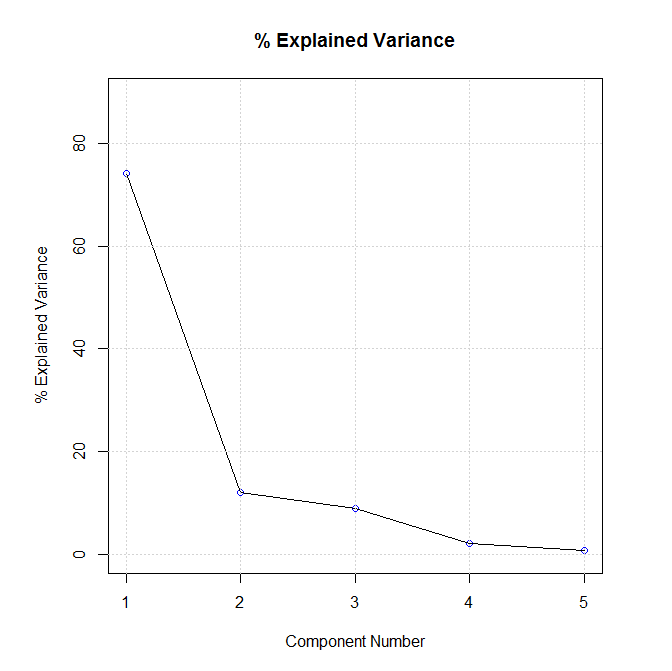

Supplement: Supplementary file 1 [file foods-08-00466-s001.zip › SUPPLEMENTARY MATERIAL/Figure S1.docx]
